# The Origin and Activities of IgA1-Containing Immune Complexes in IgA Nephropathy

**DOI:** 10.3389/fimmu.2016.00117

**Published:** 2016-04-12

**Authors:** Barbora Knoppova, Colin Reily, Nicolas Maillard, Dana V. Rizk, Zina Moldoveanu, Jiri Mestecky, Milan Raska, Matthew B. Renfrow, Bruce A. Julian, Jan Novak

**Affiliations:** ^1^Department of Microbiology, University of Alabama at Birmingham, Birmingham, AL, USA; ^2^Department of Immunology, Faculty of Medicine and Dentistry, Palacky University and University Hospital, Olomouc, Czech Republic; ^3^Department of Medicine, University of Alabama at Birmingham, Birmingham, AL, USA; ^4^Université Jean Monnet, Saint Etienne, France; ^5^PRES Université de Lyon, Lyon, France; ^6^Department of Biochemistry and Molecular Genetics, University of Alabama at Birmingham, Birmingham, AL, USA

**Keywords:** IgA, nephropathy, immune complexes, autoantibodies, complement C3

## Abstract

IgA nephropathy (IgAN) is the most common primary glomerulonephritis, frequently leading to end-stage renal disease, as there is no disease-specific therapy. IgAN is diagnosed from pathological assessment of a renal biopsy specimen based on predominant or codominant IgA-containing immunodeposits, usually with complement C3 co-deposits and with variable presence of IgG and/or IgM. The IgA in these renal deposits is galactose-deficient IgA1, with less than a full complement of galactose residues on the *O*-glycans in the hinge region of the heavy chains. Research from the past decade led to the definition of IgAN as an autoimmune disease with a multi-hit pathogenetic process with contributing genetic and environmental components. In this process, circulating galactose-deficient IgA1 (autoantigen) is bound by antiglycan IgG or IgA (autoantibodies) to form immune complexes. Some of these circulating complexes deposit in glomeruli, and thereby activate mesangial cells and induce renal injury through cellular proliferation and overproduction of extracellular matrix components and cytokines/chemokines. Glycosylation pathways associated with production of the autoantigen and the unique characteristics of the corresponding autoantibodies in patients with IgAN have been uncovered. Complement likely plays a significant role in the formation and the nephritogenic activities of these complexes. Complement activation is mediated through the alternative and lectin pathways and probably occurs systemically on IgA1-containing circulating immune complexes as well as locally in glomeruli. Incidence of IgAN varies greatly by geographical location; the disease is rare in central Africa but accounts for up to 40% of native-kidney biopsies in eastern Asia. Some of this variation may be explained by genetically determined influences on the pathogenesis of the disease. Genome-wide association studies to date have identified several loci associated with IgAN. Some of these loci are associated with the increased prevalence of IgAN, whereas others, such as deletion of complement factor H-related genes 1 and 3, are protective against the disease. Understanding the molecular mechanisms and genetic and biochemical factors involved in formation and activities of pathogenic IgA1-containing immune complexes will enable the development of future disease-specific therapies as well as identification of non-invasive disease-specific biomarkers.

## Introduction

### Diagnosis of IgA Nephropathy

**Figure 1:**
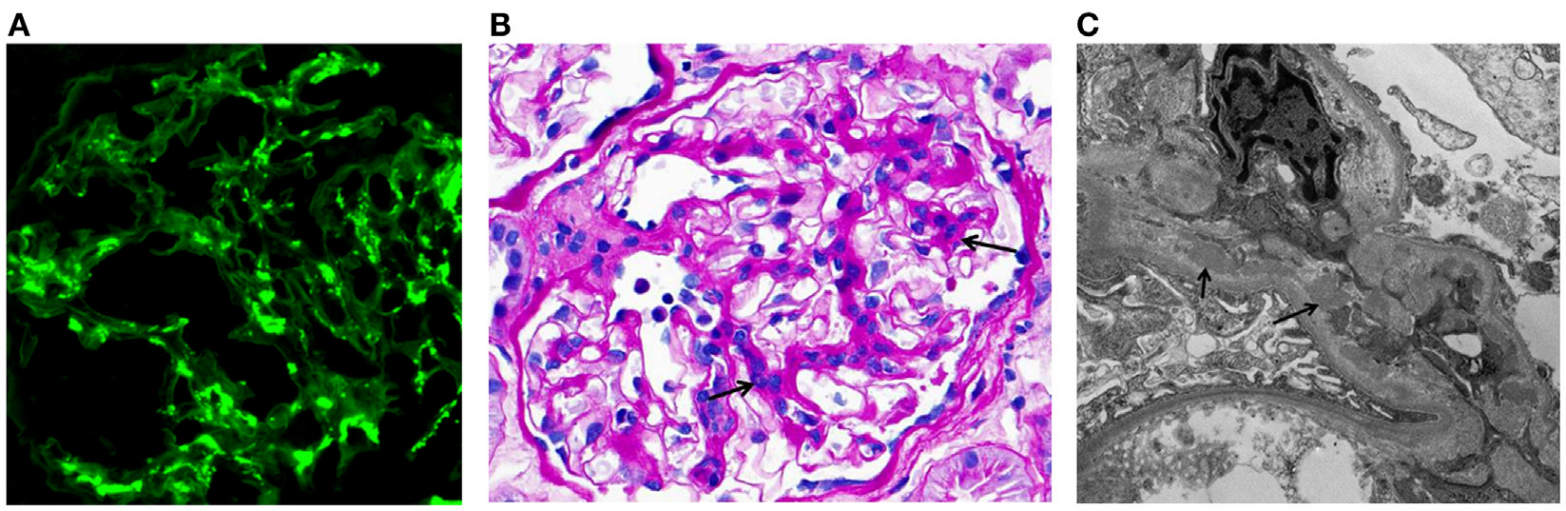
**Examples of immunofluorescence-, light-, and electron-microscopy features of renal biopsy specimens from patients with IgAN**. **(A)** Immunofluorescence staining for IgA in a kidney biopsy specimen from a patient with IgAN showing mesangial staining. **(B)** Periodic acid–Schiff staining of a kidney biopsy specimen from a patient with IgAN. Arrows indicate mesangial expansion and hypercellularity. **(C)** Electron micrograph of kidney biopsy specimen from a patient with IgAN. Arrows point to examples of electron-dense material representative of mesangial and paramesangial immune complex deposits. Images are courtesy of Dr. Huma Fatima (**B,C**) and Dr. Lea Novak (A), Department of Pathology, UAB.

IgA nephropathy (IgAN) is currently recognized as the most common primary glomerulonephritis in the world and is a frequent cause of end-stage renal disease. The diagnosis is established by immunofluorescence examination of cortical renal tissue that shows IgA as the dominant or codominant immunoglobulin in glomeruli (Figure 1A) (1, 2). Complement protein C3 is frequently present, often accompanied by IgG, IgM, or both. Confocal microscopy shows colocalization of these proteins, consistent with the presence of immune complexes. Light microscopy findings usually include mesangial hypercellularity and increased mesangial matrix (Figure 1B). Electron microscopy shows electron-dense deposits consistent with immune complexes in the mesangial and paramesangial areas (Figure 1C), occasionally with subepithelial or subendothelial deposits. In 2009, the Oxford classification of IgAN was published. This classification was put forth by an international group of nephrologists and renal pathologists to standardize pathologic findings and ascertain those that predict disease progression. Ultimately, four pathologic features were identified as being of prognostic value, independent of clinical data: mesangial hypercellularity, segmental glomerulosclerosis, endocapillary hypercellularity, and tubular atrophy/interstitial fibrosis. This classification allows the pathologist to give a score for each of these features that correlates to clinical outcome. Most cases used to develop the Oxford classification did not have significant crescents or necrosis, and therefore, neither of these findings was included in the assessment (3, 4).

### Clinical Presentation and Pathology of IgA-Related Nephritis

#### Primary IgA Nephropathy

IgA nephropathy may affect children as young as 4 years of age. The most common clinical presentation in children is visible hematuria accompanying a febrile illness, frequently an infection of the upper respiratory tract. Among adults, visible hematuria is much less common (extremely rare beyond age 40 years), and typical manifestations include microscopic hematuria, proteinuria, hypertension, and variable degrees of chronic kidney disease (5, 6). The gender distribution differs geographically, with a 2–3:1 male-to-female ratio in North America compared with a 1:1 ratio in Asia (6). About 5–8% of patients have a first- or second-degree relative with biopsy-proven IgAN or urinary abnormalities, suggesting that genetic factors influence the expression of disease. The prevalence of disease varies greatly between different regions of the world. East Asia has the highest rates, whereas the disease is rare in central Africa (7). A recent genome-wide association study (GWAS) found that the frequency of risk alleles in regional populations correlated with disease prevalence (8). The true prevalence of IgAN is impossible to establish because the diagnosis currently requires a kidney biopsy and criteria for undertaking the invasive procedure vary widely. Furthermore, IgAN is frequently subclinical, as evidenced by a study in Finland that found IgAN in 1.3% of autopsies of persons who had committed suicide or died violently (9) and a Japanese study in which biopsies of renal allografts at implantation showed IgAN in 1.6% of cases (10). Although clinical series published shortly after the discovery of IgAN in 1968 indicated generally a benign clinical course, later reports with longer observation have documented progression to end-stage renal disease in 14–39% of patients by 20 years after diagnosis (5). On the one hand, for those who undergo renal transplantation, the disease recurs in about 50% of allografts by 10 years after engraftment (11). On the other hand, the IgA immune deposits clear within several weeks from allografts with subclinical disease at the time of transplantation (12).

#### Henoch–Schönlein Purpura with Nephritis

Henoch–Schönlein purpura (HSP) is the most common vasculitis in childhood with an incidence of 6–24 per 100,000 children per year (13, 14). Extrarenal involvement includes skin (palpable purpura), gastrointestinal tract (abdominal pain and bloody diarrhea), and musculoskeletal system (arthritis and arthralgia) (15). Renal disease affects a minority of HSP patients and typically manifests as hematuria and proteinuria about 6 weeks after the appearance of purpura. Histological features of HSP with nephritis are pathologically indistinguishable from those of IgAN, suggesting that the two entities share mechanisms of disease (16, 17). While most children with HSP with nephritis resolve their urinary abnormalities, some develop long-term kidney dysfunction and may progress to end-stage renal disease. HSP is relatively uncommon in adults, but the prognosis of HSP with nephritis is worse with increasing age. Patients with HSP with nephritis were excluded from the Oxford classification, so the prognostic value of their histopathologic findings has not been established (18).

#### Secondary IgA Nephropathy

IgA-dominant immune complex glomerulonephritis has also been described in patients with a variety of systemic diseases and is thought to be a secondary manifestation. The pathogenesis behind these associations has not been elucidated, but several theories have been proposed (19). In patients with cirrhosis due to alcohol abuse or chronic infection with hepatitis C virus, glomerular IgA is thought to result from decreased clearance of the immunoglobulin by hepatocytes (20, 21). Patients with inflammatory bowel disease or celiac disease may be exposed to increased loads and variety of antigens due to impaired integrity of the gastrointestinal mucosa, inciting increased synthesis of IgA as well as abnormalities of the IgA immune system (20). Finally, chronic infections, such as those caused by staphylococci, may increase production of pathogenic IgA (20).

## Circulating IgA-Containing Immune Complexes in IgA Nephropathy and Henoch–Schönlein Purpura Nephritis

Considerable evidence has suggested that mesangial immunodeposits in IgAN are derived from IgA-containing circulating immune complexes: (1) disease recurs in about 50% of IgAN patients after kidney transplantation (22–26); (2) immune deposits clear within weeks in kidney from a person with subclinical IgAN after transplantation into a patient with non-IgAN renal disease (12); (3) blood levels of IgA and IgA-containing immune complexes are elevated in many patients with IgAN (25, 27–32); and (4) circulating complexes and mesangial deposits share idiotypic determinants (33), although a disease-specific idiotype has not been identified (34). Thus, circulating immune complexes likely play a key role in IgAN, and kidneys are “innocent bystanders.”

The apparent key role of IgA-containing immune complexes in IgAN and HSP with nephritis has been supported by data from several other studies. Circulating immune complexes with IgA and C3 are elevated in approximately one half of patients with IgAN (28). Moreover, serum levels of IgA-containing immune complexes in patients with IgAN correlate to clinical and histological activity, such as magnitude of microscopic hematuria and percentage of glomeruli with florid crescents (27, 35). In IgAN, hematuria is typical and often includes episodes of macroscopic bleeding that coincide with mucosal infections, including those of the upper respiratory tract and digestive system. These and other observations, and the fact that IgA in immunodeposits is polymeric, have indicated potential involvement of mucosal system [for review, see Ref. (36)].

Circulating immune complexes containing IgA are present in serum of healthy individuals and patients with diseases other than IgAN. Although immune complexes in such subjects may form, for example, due to binding of IgA antibodies to food or microbial antigens, it was shown for patients with IgAN that the microbial and food antigens are not substantial components of IgA-containing glomerular immunodeposits (37).

## IgA1 Structure, Production, and Metabolism

### Structure and Glycosylation of IgA1 and Pathogenesis of IgA Nephropathy

**Figure 2:**
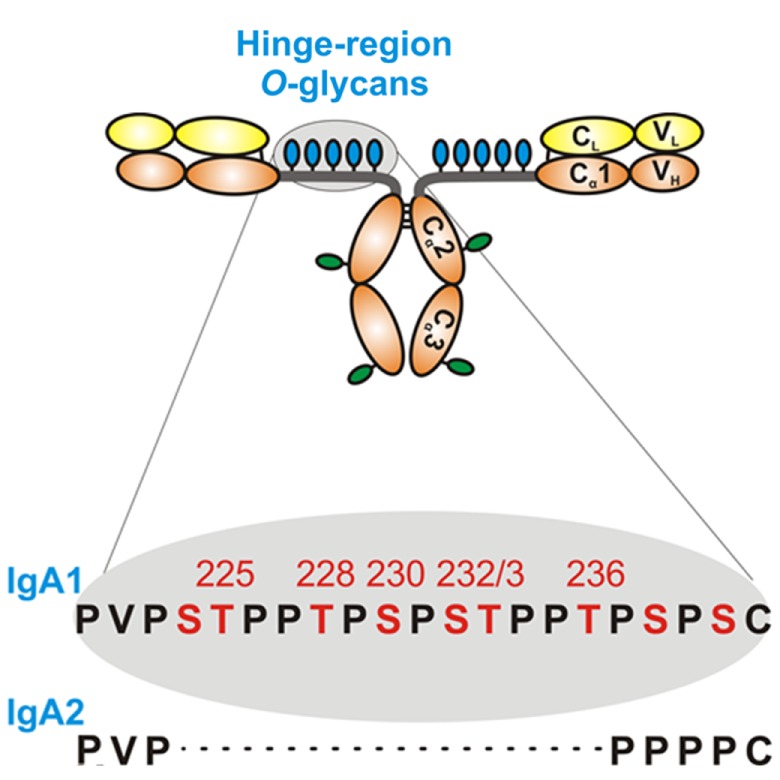
**Hinge-region glycosylation of human IgA1 and comparison of amino-acid sequences of human IgA1 and IgA2**. Human IgA1 has nine Ser (S) and Thr (T) amino-acid residues in the hinge-region segment (between constant regions C1 and C2 of the heavy chains). Usually, three to six clustered *O*-glycans are attached per hinge region. IgA2 hinge region is shorter compared to that of IgA1, does not have Ser and Thr residues and, thus, IgA2 does not have *O*-glycans. Moreover, each IgA1 heavy chain has two *N*-glycans, one in the C2 domain and the second in the tailpiece portion of the C3 domain.

**Figure 3:**
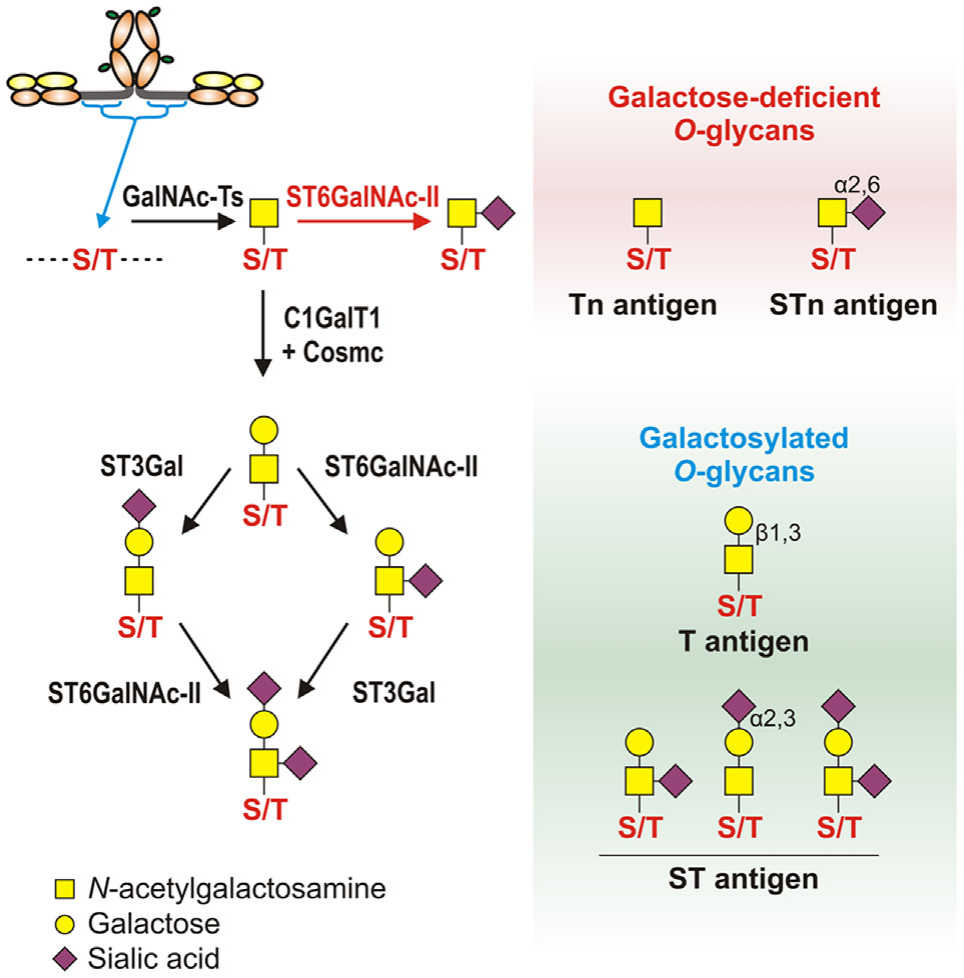
**Pathways of *O*-glycosylation of IgA1 hinge region, including galactose-deficient and galactosylated *O*-glycans**. *Left panel*: *O*-glycosylation of IgA1 hinge region occurs in the Golgi apparatus and begins with attachment of *N*-acetylgalactosamine (GalNAc) to Ser or Thr by an enzyme of UDP-GalNAc:polypeptide GalNAc-transferases family (GalNAc-Ts). In patients with IgAN, some terminal GalNAc residues may be prematurely sialylated by GalNAc α2,6-sialyltransferase (ST6GalNAc) (red arrow); this step prevents addition of galactose (the glycan thus remains galactose-deficient). In healthy individuals, GalNAc-α-Ser/Thr residue can be normally modified by addition of galactose, catalyzed by UDP-galactose:GalNAc-α-Ser/Thr β1,3-galactosyltransferase (C1GalT1); stability of C1GalT1 requires molecular chaperone Cosmc. Galβ1,3-GalNAc structures may be further modified by addition of sialic acid to galactose residues through the activity of Galβ1,3-GalNAc α2,3-sialyltransferase (ST3Gal) and/or to GalNAc residues through the activity of ST6GalNAc. *Right panel*: galactose-deficient *O*-glycans consist of terminal GalNAc, also known as Tn antigen, or GalNAc with α2,6-linked sialic acid, also known as STn antigen. Galactosylated *O*-glycans are disaccharides consisting of galactose and GalNAc (Galβ1,3-GalNAcα1-O-Ser/Thr, also known as T antigen) and may be modified by sialic acid (also known as ST antigen). T antigen does not carry sialic acid, but ST antigen has sialic acid attached to galactose and/or GalNAc.

Humans have two IgA subclasses, IgA1 and IgA2. IgA1 contains *O*-glycans attached to Ser or Thr, usually three to six, in the hinge region (HR) of the heavy chains (Figure 2). IgA1 HR has nine Ser and Thr amino-acid residues; those are missing in IgA2 HR and, thus, IgA2 does not have *O*-glycans (Figure 2). In normal human serum IgA1, HR glycoforms with four and five glycans are the most common [for review, see Ref. (38)]. Each heavy chain of IgA1 also contains two *N*-glycans, one in the CH2 domain (Asn263) and the second in the tailpiece portion (Asn459) (39, 40). Normal human circulatory IgA1 usually has core 1 *O*-glycans consisting of *N*-acetylgalactosamine (GalNAc) with β1,3-linked galactose. One or both saccharides can be sialylated, galactose with α2,3-linked and GalNAc with α2,6-linked sialic acid (Figure 3, right panel). The composition of the *O*-glycans on normal serum IgA1 is variable; prevailing forms include the GalNAc-galactose disaccharide and its mono- and di-sialylated forms (41–44). Normal serum IgA1 had been thought to contain little or no galactose-deficient *O*-glycans (44), but it is now considered that some terminal or sialylated GalNAc is likely present even in healthy individuals (45).

Analysis of IgA in patients with IgAN revealed that abnormal *O*-glycosylation is a key step directing IgA1 immune complex formation and glomerular deposition (31, 46–52). The accumulated data suggest that circulating complexes in patients with IgAN contain galactose-deficient IgA1 (Gd-IgA1) (31, 49, 50, 53) and that the IgA in the mesangial deposits is exclusively of IgA1 subclass (54) and is enriched for Gd-IgA1 glycoforms (55, 56). Further insight about a relationship between Gd-IgA1 and nephritis has come from other observations: (1) Gd-IgA1 (57) and IgA–IgG circulating immune complexes (58) are in sera of patients with HSP with nephritis but not in sera of patients with HSP without nephritis and (2) patients with IgA1 myeloma have high circulating levels of IgA1, but only those with aberrantly glycosylated IgA1 develop immune complex glomerulonephritis (59, 60).

Human serum IgA, predominantly IgA1 with a small contribution of IgA2, is >90% in monomeric form and <10% in polymeric form, and a small fraction is bound in circulating immune complexes (61). Serum IgA1 is rapidly catabolized by hepatocytes (see below for more details) and thus has a short half-life (~5 days) (62). Hepatocytes express asialoglycoprotein receptor (ASGP-R) (63, 64) that binds IgA1 and other glycoproteins through terminal galactose or GalNAc residues (63–65). Gd-IgA1 remains in the circulation for a prolonged period of time (66). Galactose deficiency in itself should not hinder catabolism of IgA1 molecules because ASGP-R can recognize terminal GalNAc (65). However, if sialic acid is linked to GalNAc or IgA1 is bound by an antibody, then such IgA1 cannot be recognized by the receptor (53, 67). Serum Gd-IgA1 is bound primarily within immune complexes (31, 49). The large size of these complexes likely precludes entry into the space of Disse through relatively small endothelial fenestrae (68), hence preventing their hepatic clearance from the circulation (69–71). Immune complexes then deposit in the mesangium after passing through larger fenestrae in glomerular capillaries (36, 72–74). This postulate is consistent with observations that, in animals, large-molecular-mass immune complexes induce more severe glomerular lesions than do small complexes (75).

### Approaches for Analysis of IgA1 Aberrant *O*-Glycosylation

#### Initial Approaches

Abnormality of IgA1 *O*-glycans in patients with IgAN was first indicated by an observation of reduced reactivities of IgA1 with jacalin, a lectin-binding galactose-GalNAc disaccharide (46). Based on additional research, defective galactosylation of *O*-glycans of IgA1 molecules was proposed as an etiopathogenic factor in IgAN (47). Various lectin-binding assays were used to examine the presence of terminal galactose on *N*-glycans of purified serum IgG and IgA1 and *O*-glycans of IgA1 and C1 inhibitor (76). Serum IgA1 of patients with IgAN *vs*. controls had less galactose on GalNAc, whereas the glycosylation of C1 inhibitor did not exhibit this difference in glycosylation. Another study compared *O*-glycosylation of serum IgA1 and IgD, a second immunoglobulin with *O*-glycans, in patients with IgAN and healthy controls and found aberrant *O*-glycosylation only on IgA1 from patients with IgAN (77). Together, these data suggested that patients with IgAN had galactose-deficient *O*-glycans uniquely on circulatory IgA1 (78). These findings were confirmed using a panel of lectins, including that from *Helix aspersa*, specific for terminal GalNAc (31). Moreover, Gd-IgA1 was present in complexes with IgG, leading to speculation that formation of these complexes may reduce the rate of elimination of immune complex-bound IgA1 and lead to elevated serum levels of Gd-IgA1 (31, 79). These findings explained why serum levels of IgA1-containing immune complexes of patients with IgAN and HSP with nephritis are higher than those in healthy controls and, furthermore, why IgA1-containing immune complexes frequently contain also IgG (27, 31).

Additional assessments of IgA1 *O*-glycans used different analytical approaches, including lectins recognizing different *O*-glycans on intact IgA1 molecules, monosaccharide compositional analysis by gas–liquid chromatography (LC), mass spectrometric analysis of isolated *O*-glycosylated hinge-region glycopeptides, Edman sequencing, and separation and identification of free *O*-glycans released from IgA1 (31, 36, 41–44, 47, 70, 78, 80–96). Each technique presented advantages and disadvantages. For example, lectin ELISA allows high-throughput analyses in a quantitative manner (89, 90, 94) but does not provide information on sites of attachment and heterogeneity in the HR, whereas the more cumbersome methods of mass spectrometry will provide molecular-level details.

#### Mass Spectrometry

By the mid-1990s, mass spectrometry became the standard tool for analysis of IgA1 *O*-glycosylation, revealing variably *O*-glycosylated HR glycoforms. Two IgA1 HR glycopeptides containing four or five *O*-glycan chains were identified by MALDI-TOF mass spectrometry (97). Later analyses used normal serum IgA1 *O*-glycopeptides (98, 99), pooled serum of patients with IgAN (100), IgA1 isolated from pooled renal biopsies (56), and tonsillar IgA1 (101). Mass spectrometric analysis showed differences in HR *O*-glycopeptides of IgA1 from patients with IgAN *vs*. healthy controls (usually serum IgA1), consistent with less galactosylation in patients with IgAN (56, 100, 101). IgA-specific proteases that released IgA1 HR fragments of different lengths provided new tools for generating IgA1 HR *O*-glycopeptides for analysis (84). A method for direct localization of sites of *O*-glycan attachment in IgA1 myeloma protein was developed by the use of electron capture dissociation (ECD) tandem mass spectrometry (MS/MS) (102). For the first time, individual sites of *O*-glycan attachment were directly identified for individual IgA1 HR glycoforms. These data confirmed Thr225, Thr228, Ser230, Ser232, and Thr236 as sites of glycan attachment in a single IgA1 HR *O*-glycoform with five *O*-glycans and Thr225, Thr228, Ser230, and Ser232 as the sites of glycan attachment in two HR *O*-glycoforms with four *O*-glycans (102). The ability to localize all sites of glycosylation in a single IgA1 HR species expanded the possibilities of defining the heterogeneity and aberrant glycosylation of IgA1 from patients with IgAN.

Renfrow et al. pursued the *O*-glycan analysis of three distinct IgA1 myeloma proteins using reversed-phase LC separation of IgA1 *O*-glycopeptides and ECD fragmentation of a larger IgA1 HR tryptic fragment and the second fragment released by IgA-specific proteases (103), demonstrating the utility of high-resolution mass spectrometry. In 2010, they reported the complete localization of all sites of *O*-glycosylation in the six most abundant IgA1 *O*-glycoforms of an IgA1 myeloma protein (104). Three distinct IgA1 HR proteolytic fragments were analyzed, and the pattern of glycopeptides for each proteolytic fragment was assigned a relative distribution based on a label-free relative quantitative method developed for *N*-glycopeptides (105). Specific sites of galactose deficiency have been expressed as a percentage of the total distribution of all observed *O*-glycoforms. ECD and a newer ECD-type fragmentation method, electron transfer dissociation (ETD), were used to localize sites of *O*-glycan attachment with LC–MS/MS (103, 106). In 2012, a new type of heterogeneity was identified, representing IgA1 *O*-glycopeptide isomers, *i.e*., equally *O*-glycosylated IgA1 HRs with different sites of attachment (45), involving Ser230, Thr233, and Thr236 sites. With these 2010 and 2012 studies, the sites of attachment indicated a semi-ordered synthesis of the clustered IgA1 *O*-glycans and not a series of random attachments. Hopefully, these approaches will elucidate the structural basis of abnormal *O*-glycosylation of IgA1 in IgAN and provide clues as to whether specific isomers are associated with the clinical expression or course of the disease.

### IgA Molecular Forms and IgA Production and Catabolism

#### IgA Subclasses

Molecules of monomeric IgA contain two α1 or two α2 chains, linked by inter α-chain disulfide bridges and two κ or two λ chains. A distinguishing feature of polymeric IgA, irrespective of its dimeric or tetrameric form, is the presence of a single molecule of joining (J) chain incorporated into polymeric IgA within IgA-producing cells (107). The role of J chain in the process of polymerization of IgA remains unresolved; polymeric IgA and IgM molecules devoid of J chain have been described [for review, see Ref. (108)]. Human α1 and α2 chains as well as α chains from other species comprise one variable- and three constant-region domains, each containing ~110 amino acids. Although comparable in its general structure to the γ chains of IgG, there are several important structural differences that are characteristic of α chains. These differences include the unique HR between Cα1 and Cα2 domains, the extension of the C terminus of the α over γ chains by 18 amino acids essential for the J chain binding and polymerization, and the glycan moieties characteristic of the α1 and α2 chains (107). All three constant-region domains of α1 and α2 chain have 90–98% primary structure homology; the difference is restricted to the HR and allotype-associated sequences in IgA2 molecules. There are 17 Cys residues that participate in the intradomain and interchain disulfide bridges. In polymeric IgA, the penultimate Cys residue of the α chain tailpiece is involved in the binding of J chain and formation of polymers. This small glycosylated peptide contains ~137 amino acids (107). The major structural difference between α1 and α2 heavy chains occurs in the HR that consists of 26 and 13 amino-acid residues in α1 and α2 chains, respectively. The additional 13 amino-acid residues in IgA1 HR consist of repeated sequences of Pro, Ser, and Thr residues. By its general structure, HR is reminiscent of mucin molecules.

The total circulating pools of IgA1 and IgA2 are 101 ± 26 and 14 ± 4 mg/kg body weight, respectively (109). Approximately 55% of total IgA is in the intravascular compartment; the remainder is in interstitial fluid. These data do not include IgA produced in mucosal tissues and selectively transported into the external secretions (secretory IgA). However, IgA from mucosal tissues contributes only small quantities to the circulatory pool (110).

IgA1 HR is a target of IgA-specific proteases produced by pathogenic bacteria, such as *Haemophilus influenzae*, *Streptococcus pneumoniae*, *Neisseria meningitides*, and *Neisseria gonorrhoeae* (111). Furthermore, the extended IgA1 HR confers greater flexibility of Fab “arms” (107) and facilitates interactions with antigens. The tailpiece of α chains and of μ chains of IgM contains a Cys residue to which J chain is attached. The presence of J chain in polymeric IgA and IgM is essential for the binding of polymeric immunoglobulin receptor (112). Not all polymeric IgA and IgM molecules contain J chain. For example, hexametric IgM produced in small quantities at the early phase of the immune response is devoid of J chain. Similarly, it appears that various human polymeric IgA myeloma proteins display variable J chain content. J chain is produced not only in plasma cells synthesizing polymeric IgA or IgM but also in IgG-, IgD-, or light-chain-producing multiple myeloma cells from mucosal tissues and bone marrow (107). The presence of J chain-containing polymeric IgA in circulating immune complexes and in mesangial deposits of IgAN patients suggests a mucosal origin of IgA1; however, the possibility that such polymeric IgA1 molecules are produced in the bone marrow of IgAN patients has been proposed (113). Further studies are needed to address this point.

Several investigators noted the effect of *O*-glycan heterogeneity on the propensity of some IgA1 glycoforms to aggregate under laboratory conditions using elevated temperatures (114). Moreover, non-galactosylated glycoforms of IgA1 exhibited binding with proteins of extracellular matrix (115). The authors of these studies suggested that IgA1 *O*-glycans played a protective role against aggregation and adhesion and that the underglycosylation of the IgA1 molecule may be involved in the non-immunologic glomerular accumulation of IgA1. It is not clear whether glomerular deposition of IgA1 that is not bound in complexes would lead to pathological consequences, *i.e*., mesangial proliferation and matrix expansion, and under what circumstances, and whether such a mechanism may play a role in the postulated heterogeneity of IgAN (116).

Immunohistochemical studies and results from short-term culture experiments of human tissues supported the above-described distribution of the form of IgA (polymeric or monomeric) and the isotype (IgA1 or IgA2) in several fluids that parallels the distribution of cells in various tissues and organs. Measurements of antigen-specific antibodies in individual external secretions mirrored the distribution of IgA1- or IgA2-producing cells in the corresponding mucosal tissues (107, 117). Furthermore, IgA-producing cells abundant in mucosal tissues secrete polymeric IgA that is efficiently transported through epithelial cells by a receptor-mediated mechanism into external secretions (112). Nevertheless, the contribution and location of polymeric IgA-producing cells to the circulating pool of IgA remain to be determined (107, 109).

The tissue origin of polymeric Gd-IgA1 bound in the circulating immune complexes and in mesangial immunodeposits of patients with IgAN is unclear. On the one hand, it is assumed that because of its polymeric character, Gd-IgA1 originates in mucosal tissues of the respiratory and/or gastrointestinal tracts. On the other hand, it is possible that the IgA-producing cells in the bone marrow may secrete, in addition to the dominant monomeric IgA1, also small quantities of polymeric IgA1 as a consequence of infection. The initial IgA responses to an infection or immunization, irrespective of the systemic or mucosal route of vaccination, are dominated by polymeric IgA in serum and secretions [for review, see Ref. (118–120)].

Studies of the association of naturally occurring or immunization-induced serum and secretory IgA antibodies to different types of antigens provided several highly relevant findings (107, 109). Antibodies specific for protein-, glycoprotein-, and virus-derived antigens (*e.g*., influenza and HIV) are dominantly of the IgA1 subclass; in contrast, antibodies against polysaccharides, lipopolysaccharides, and teichoic acid are associated with the IgA2 subclass. Notably, systemic or mucosal immunization with influenza virus vaccine induces a mainly polymeric IgA1 response in serum; polymeric IgA2-dominant responses are detected in individuals immunized with polysaccharide vaccines (107, 109). Thus, the type of the antigen substantially influences the IgA subclass-associated response. Some studies showed that patients with IgAN had reduced in IgA1 responses to challenges with some antigens (121, 122), whereas another study observed differential *O*-glycosylation of IgA1 antibodies against mucosal *vs*. systemic antigens (120). Moreover, some investigators have found secretory IgA1 (with secretory component) in renal deposits (123, 124) or polymeric IgA1 (125), suggesting that this IgA1 was generated during a mucosal immune response (126). It is not clear whether secretory IgA1, regardless of its *O*-glycosylation pattern, may be a major driver of the pathogenesis of IgAN.

#### IgA1-Producing Cells

The macroscopic hematuria associated with upper respiratory tract infections in patients with IgAN suggests that the synpharyngitic hematuria may reflect an inflammatory environment conducive to driving renal complications (127). IgA produced in the mucosal compartments is polymeric, the predominant form of Gd-IgA1 (128). Thus, circulatory Gd-IgA1 may originate from mucosal tissues, and local infections may accentuate Gd-IgA1 production. This concept is the subject of ongoing research that may elucidate mechanisms, which are responsible for increased levels of circulatory Gd-IgA1.

IgA1 production at mucosal tissues from resident IgA1-producing cells serves several functions; in this review, we will focus on mechanisms of aberrant IgA1 *O*-glycosylation in patients with IgAN. The Japanese Society of Nephrology now recommends tonsillectomy for treatment of IgAN, as tonsillectomy in combination with glucocorticoid pulse therapy improved renal outcomes in many patients with IgAN and macroscopic hematuria (129). However, a benefit of tonsillectomy on disease progression was not found in European cohorts (130), which could be due to genetic differences or early screening that is routinely done in Japan. Recent data suggest that B cells isolated from tonsils of patients with IgAN exhibit increased IL-4 and IFNγ production upon exposure to hemolytic streptococci and lipopolysaccharides when compared to tonsillar B cells from controls (131). Increased numbers of memory B cells were found in tonsils (5.7 *vs*. 1.8%) and peripheral blood (4.9 *vs*. 0.9%) of IgAN patients compared to controls; this finding correlated with proteinuria (*r* = 0.81) (132). Moreover, patients with IgAN after tonsillectomy had fewer peripheral blood memory B cells (4.9% regressed to 1.1%) (132). These studies highlight the role of inflammation and the importance of the mucosal-circulatory connection in patients with IgAN.

Other studies revealed increases in TLR9 and B-cell-activating factor (BAFF) mRNA expression in peripheral blood mononuclear cells as well as increased serum levels of BAFF protein (133). Mice overexpressing human BAFF develop a commensal microbiota-dependent IgA-associated nephropathy (134, 135). BAFF induces class-switch recombination in B cells and may drive the circulatory IgA1 levels in patients with IgAN (136). Moreover, more L-selectin was found in B and T cells derived from the circulation of patients with IgAN (137, 138). Together, these data suggest a proinflammatory state of B cells in patients with IgAN. This finding corroborates *in vitro* data, showing that certain cytokines can enhance production of Gd-IgA1 (139).

To study molecular mechanisms of production of Gd-IgA1, peripheral blood mononuclear cells and tonsillar B cells were isolated from IgAN patients and controls, and Epstein–Barr virus (EBV)-immortalized cells were generated. From these mixed cell lines, IgA1-producing cells were isolated through limiting dilution subcloning. Analysis of IgA1 secreted by these cell lines derived from blood of patients with IgAN showed enhanced production of Gd-IgA1 when compared to controls. The degree of galactose deficiency of IgA1 secreted by EBV-immortalized B cells corresponded to the serum Gd-IgA1 levels from the corresponding donors, indicating that glycosylation of IgA1 and Gd-IgA1 production had not been altered by EBV immortalization (140). These cell lines provide a new tool for studies of biosynthesis of Gd-IgA1 (93).

#### Signaling in IgA1-Producing Cells

As noted above, patients with IgAN often exhibit macroscopic hematuria associated with mucosal infections. These infections may be associated with increased production of IgA and Gd-IgA1 (141). The exacerbation of kidney damage associated with acute infection/inflammation in patients with IgAN may be transient or permanent, and it indicates a connection with activated immune system (127). Increased levels of markers of inflammation, such as IL-6 and soluble vascular cell adhesion molecule-1 (sVCAM-1), have been found in the blood of patients with IgAN (142, 143). Some proinflammatory cytokines, such as IL-6 and leukemia inhibitory factor (LIF), increase production of Gd-IgA1 in B cells from patients but not controls (139). In IgA1-producing cells from patients with IgAN *vs*. healthy controls, IL-6 showed increased and prolonged activation of STAT3 (144). As STAT3 is the canonical transcription factor of IL-6 and other cytokines, changes in signaling and transcription driven by STAT3 may have an important role in Gd-IgA1 production (145).

Production of Gd-IgA1 in patients with IgAN has been linked to aberrant expression and activities of specific glycosylation enzymes in the Golgi apparatus for normal *O*-glycosylation (146, 147). Galactose deficiency of IgA1 *O*-glycans can be due to a reduced rate of galactosylation or premature sialylation that would prevent addition of galactose. Further dysregulation by IL-6 of the corresponding enzymes (see Mechanisms and Pathways Involved in Production of Aberrantly Glycosylated IgA1 for details) involved in these processes was observed (139), but the detailed mechanism that leads to these changes is unknown.

**Figure 4:**
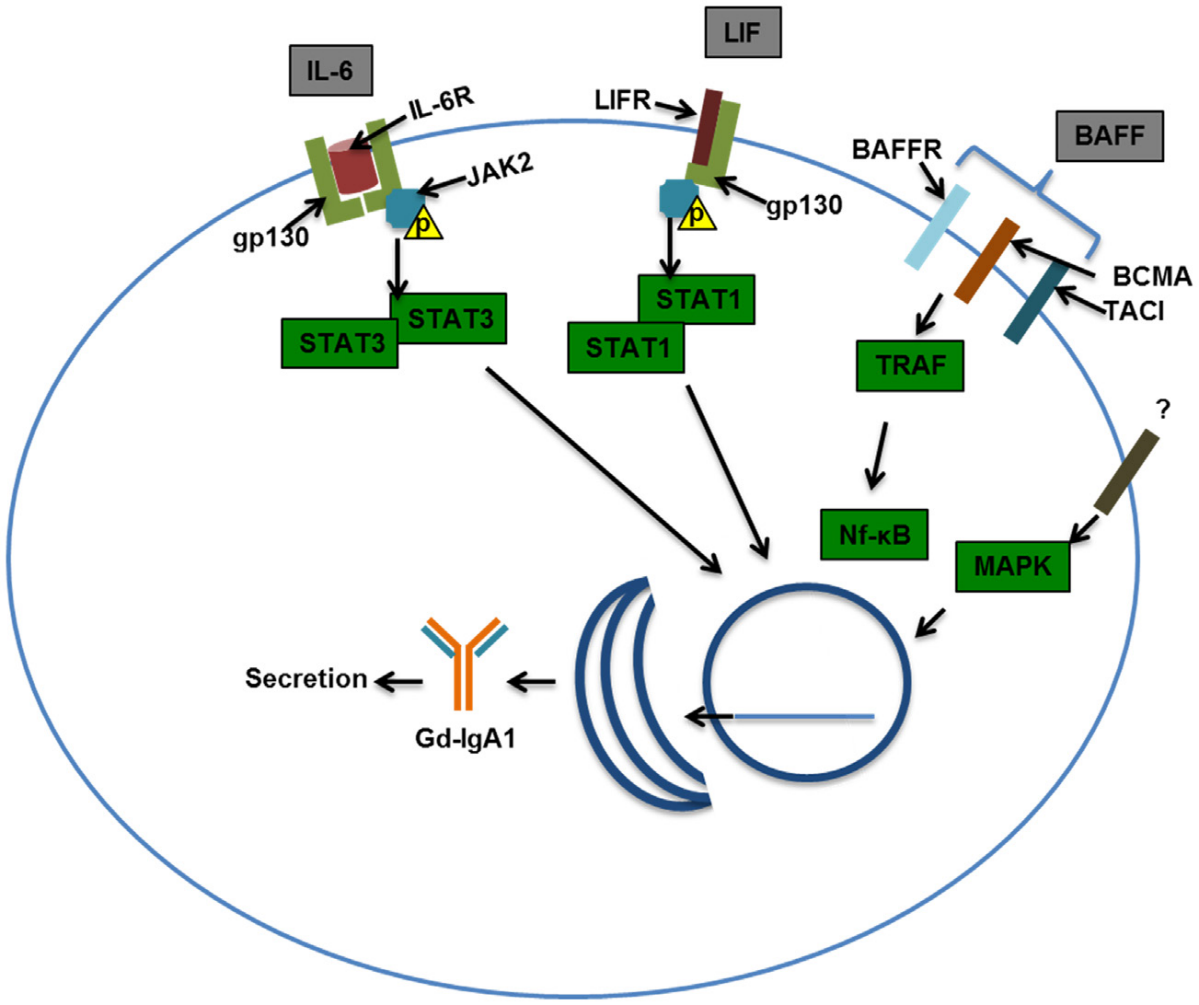
**Examples of signaling pathways that may affect production of galactose-deficient IgA1**. Interleukin-6 (IL-6) binds to the IL-6 receptor (IL-6R) and through co-receptor gp130 (glycoprotein 130) activates Janus kinase 2 (JAK2), which in turn phosphorylates signal transducer and activator transcription 3 (STAT3), leading to its dimerization and nuclear translocation. Leukemia inhibitory factor (LIF) binds gp130 and LIF receptor (LIFR) that also activates JAK2, leading to STAT1/3 activation and nuclear translocation. B-cell-activating factor (BAFF) can bind multiple receptors, BAFF receptor (BR), B-cell maturation antigen (BCMA), and transmembrane activator and calcium-modulating/cyclophilin ligand protein (TACI). Activation of TNF-receptor-associated factor (TRAF) by TACI, BAFFR, and BCMA leads to NF-*k*B activation and nuclear translocation. Mitogen-activated protein kinase (MAPK) activation has been shown to affect *O*-glycosylation and thus was included in this scheme. Nuclear translocation of the STATs and NF-*k*B may be important in driving production of Gd-IgA1 in IgA1-producing cells and/or maintaining viability/proliferation of these cells in patients with IgAN.

In addition to cytokines, it is also possible that other B-cell-stimulating factors may contribute to increased production of Gd-IgA1 (148). These factors, such as BAFF, may drive IgA class switching, B-cell differentiation and antibody production, and cellular proliferation (149). Such signaling ligands may share similar receptors (Figure 4). Several GWAS have implicated a locus encompassing the *APRIL* gene (*TNFSF13*) in IgAN, and serum levels of the expressed ligand were elevated in patients with IgAN (136, 150). Increased amounts of BAFF are also found in sera and tonsillar tissue of some patients with IgAN (151). Mice with BAFF overexpression exhibited a microbiota-dependent IgA-associated glomerulonephritis, further implicating B-cell activation in IgA glomerular deposition (135).

Production of Gd-IgA1 by IgA1-producing cells is enhanced in patients with IgAN, possibly through altered signaling of pathways involving STAT3. Contributing factors related to abnormal signaling could be genetic, as indicated by the increased serum levels of Gd-IgA1 in asymptomatic relatives of patients with IgAN (152). Environmental factors play a role as well, as there is a connection between infection/inflammation and disease activity. Future research is needed to define the interaction between environmental and genetic factors, and how it relates to signaling changes in IgA1-producing cells.

#### IgA Metabolism/Catabolism

IgA from the circulation is primarily catabolized in the liver (107, 110, 112). IgA bound to ASGP-R expressed on hepatocytes in the presence of Ca^2+^ is internalized, and the IgA-containing vesicles fuse with lysosomes resulting in intracellular degradation (63, 64, 153). Experiments with human IgA1 and IgA2 myeloma proteins in their monomeric or polymeric forms (62) demonstrated that in monkeys, the liver has the highest uptake of IgA. Hepatocytes compared to non-parenchymal cells were more active in the catabolism of IgA (62). Only small quantities of IgA were catabolized in the kidneys, skin, and spleen. The importance of the ASGP-R in IgA catabolism was further confirmed using a human hepatoma cell line (64). Of note, autoantibodies specific for ASGP-R have been observed in patients with autoimmune hepatitis (154). The marked species-dependent differences in the structure, transport, metabolism, and catabolism of IgA of different molecular forms must be taken into consideration in animal models of IgAN as well as the fact that different molecular dimensions of monomeric and polymeric IgA and polymeric IgA-containing immune complexes affect the catabolism and distribution of free or complexed IgA.

### Mechanisms and Pathways Involved in Production of Aberrantly Glycosylated IgA1

Normal serum IgA1 *O*-glycans consist predominantly of galactose-β1-3GalNAc dissaccharide, also known as T antigen, and its mono- or di-sialylated forms [NeuAcα2-3-galactose-β1-3GalNAc and NeuAcα2-3-galactose-β1-3(NeuAcα2-6)GalNAc, commonly described as sialyl-T (ST) antigen] (Figure 3, left panel) (44, 45). *O*-glycosylation of IgA1 HR involves multiple glycosyltransferases that add one monosaccharide at a time in a stepwise manner to a growing *O*-glycan chain. *O*-glycosylation of IgA1 takes place in the Golgi apparatus (93). *O*-glycosylation is initiated by attachment of GalNAc to Ser/Thr residues by the activity of a UDP-GalNAc:polypeptide GalNAc-transferase family (ppGalNAc-Ts), consisting of 20 members in humans (155, 156). Dominant role during IgA1 HR *O*-glycosylation was attributed to the ubiquitous GalNAc-T2 (157). Further work indicates that GalNAc-T1 and GalNAc-T11 can also initiate *O*-glycosylation of IgA1 (158). Recently, we compared transcript levels of all known human GalNAc-Ts in IgA1-producing cells from IgAN patients and disease controls and identified significant differences for only GalNAc-T14 (159, 160). Preliminary data indicate that GalNAc-T14 could attach GalNAc to IgA1 HR and thus may contribute to the aberrant glycosylation of IgA1 (161). Interestingly, GalNAc-T14 is structurally the closest relative of GalNAc-T2 (162). Overexpression of GalNAc-T14 in IgA1-producing cells from IgAN patients could contribute to the increase in the overall number of *O*-glycans on IgA1 in IgAN patients (163).

After the initial addition of GalNAc to Ser/Thr residues, galactose is added by UDP-galactose:GalNAc-α-Ser/Thr β1,3-galactosyltransferase (C1GalT1) (164). A deficiency of C1GalT1 results in truncation of *O*-glycans (165). The biosynthesis of active C1GalT1 depends on molecular chaperone Cosmc (166, 167). Cosmc mutation(s) is associated with the expression of the terminal GalNAc and sialylated GalNAc (also called Tn and STn antigens, respectively) in various neoplastic lesions and Tn syndrome (167–169) but not in IgAN (170). Decreased levels of C1GalT1 transcript and protein activity were detected in subcloned Gd-IgA1-producing cells from IgAN patients (93). C1GalT1 deficiency is further accentuated after exposing the IgA1-producing cells to IL-6 (139). Together with the constitutionally increased activity of GalNAc-T14, IgA1-producing cells could, under local inflammatory conditions, insufficiently galactosylate GalNAc residues attached in the IgA1 HR.

Galactose-β1,3GalNAc structures are subsequently modified by attaching sialic acid from CMP-*N*-acetylneuraminic acid (CMP-NeuAc) to galactose residues by the activity of galactose-β1,3GalNAc α2,3-sialyltransferase (ST3Gal) and/or to the GalNAc residues by activity of an α2,6-sialyltransferase (ST6GalNAc) (171, 172). Neuraminidase-driven *in vitro* removal of sialic acid from IgA1 produced by EBV-immortalized cells from IgAN patients (93) and nasopharyngeal carcinoma (Dakiki cells) (146) enhanced reactivity with GalNAc-specific lectin (HAA). These studies suggested that some Tn *O*-glycans on IgA1 are capped with sialic acid (sialyl-Tn antigens) (93, 146, 173). The analysis of all known human ST6GalNAc transcripts (*ST6GALNAC1*–*6*) performed by real-time RT-PCR showed that ST6GalNAc-I, an enzyme described to be responsible for sialylation of Tn antigens, is not expressed in IgA1-producing cells; however, abundant transcription of *ST6GALNAC2* was detected. Other *ST6GALNAC* genes were transcribed either in similar extent between Gd-IgA1- and normal IgA1-producing cells (*ST6GALNAC3*, *ST6GALNAC4*, and *ST6GALNAC6*) or were not detectable (*ST6GALNAC5*) (93, 146, 174). Recombinant human ST6GalNAc-II can sialylate terminal GalNAc of IgA1 *in vitro* (174). Involvement of ST6GalNAcII in sialylation of Tn antigens on IgA1 HR was confirmed by reduced HAA reactivity with IgA1 secreted from Gd-IgA1-producing cells lines, in which ST6GalNAc-II activity was suppressed by siRNA-driven *ST6GALNAC2* knock-down (139). Subsequent *in vitro* experiments, in which α2,6-sialyltransferase and β1,3-galactosyltransferase enzymes were obtained as a Golgi extract from Gd-IgA1-producing cells, confirmed that sialylation of terminal GalNAc blocks effective galactosylation (139). Thus, premature sialylation, associated with increased transcriptional activity of *ST6GALNAC2* in Gd-IgA1-producing cells, may contribute to Gd-IgA1 production in IgAN. Sialyltransferases are localized predominantly in *trans*-Golgi compartments, but the observation that galactose-deficient sialylated GalNAc-containing IgA1 is present throughout the Golgi (93) suggested a possible abnormal relocalization of sialyltransferases toward *cis*-Golgi. This abnormality may contribute to the galactose deficiency of IgA1 *O*-glycans. However, studies of subcellular localization of individual enzymes are needed to confirm this hypothesis.

In summary, Gd-IgA1-producing cells from IgAN patients have elevated expression *GalNAc-T14* and *ST6GalNAc-II*, and decreased expression of *C1GalT1* and *Cosmc* (93, 159). As macroscopic hematuria in IgAN patients often coincides with mucosal infections, inflammation may enhance galactose deficiency of IgA1. Indeed, IL-6 and, to a lesser extent, IL-4 accentuated galactose deficiency of IgA1 secreted by cell lines from IgAN patients (139). Stimulation of cells from IgAN patients with IL-6 increased α2,6-sialyltransferase activity and decreased activity of C1GalT1, whereas IL-4 only reduced the activity of C1GalT1 (139). These experiments indicate that IgA1-producing cells from IgAN patients accentuate production of Gd-IgA1 upon stimulation with IL-6. Aberrancies in JAK–STAT signaling pathways may be involved in these processes (144).

### Genetics of Aberrant Glycosylation of IgA1

Comprehensive studies of the glycosylation abnormalities of IgA1 offered a potential phenotypic biomarker for IgAN, Gd-IgA1 (61, 69, 70, 88, 89, 175). A quantitative lectin-binding assay enabled assessment of the inheritance of Gd-IgA1 in familial and sporadic forms of IgAN (152). Elevated serum levels of Gd-IgA1 were found in most patients with IgAN, as well as many of their first-degree relatives, whereas levels in spouses were similar to those of healthy controls. Segregation analysis of Gd-IgA1 levels suggested inheritance of a major dominant gene with an additional polygenic component. The inheritance of Gd-IgA1 serum levels has been confirmed in patients with familial and sporadic IgAN (52, 176, 177), and in pediatric patients with IgAN and HSP with nephritis (178). Thus, aberrant IgA1 glycosylation is a common inherited defect that provides a unifying link in the pathogenesis of HSP with nephritis and IgAN in many populations worldwide (93, 179).

## Immune Complexes Contain Galactose-Deficient IgA1 in IgA Nephropathy

It is now well accepted that the circulation of patients with IgAN contains immune complexes consisting of Gd-IgA1 [for reviews, see Ref. (61, 180)]. Initial analyses showed that Gd-IgA1 was predominantly in large-molecular-mass fractions of serum and was associated with IgG, thus indicating a possibility that Gd-IgA1 was bound by IgG in an immune complex (31). A follow-up study confirmed that circulating immune complexes in patients with IgAN consist of polymeric Gd-IgA1 bound by IgG antibodies specific for GalNAc residues in the hinge-region *O*-glycans of IgA1 heavy chains (49).

Elevated serum levels of Gd-IgA1 are found not only in patients with IgAN but also in patients with HSP with nephritis (57, 88, 178). It is now proposed that the pathology of HSP with nephritis and IgAN is driven by glomerular deposition of large immune complexes from the circulation (6, 18). Importantly, patients with HSP without nephritis have only IgA–IgA immune complexes, whereas patients with HSP with nephritis have IgA–IgA and IgA–IgG immune complexes (58).

## Autoantibodies Against Galactose-Deficient IgA1 in IgA Nephropathy

Autoantibodies forming complexes with Gd-IgA1 in the blood of IgAN patients are predominantly of the IgG isotype (31, 181). These autoantibodies recognize HR of IgA1 with terminal GalNAc residues (31, 182). This conclusion was based on several experiments. Binding of IgG autoantibodies from serum samples of IgAN patients was tested using ELISA with several antigens: enzymatically desialylated and degalactosylated IgA1 myeloma protein (dd-IgA1), Fab fragment of Gd-IgA1 containing part of the HR with *O*-glycans (Fab-IgA1), synthetic HR peptide linked to bovine albumin (HR-BSA), and a synthetic HR glycopeptide with three GalNAc residues linked to BSA (HR-GalNAc-BSA). Binding to dd-IgA1 and Fab-IgA1 was significantly higher for IgG from sera of patients with IgAN than that for IgG from sera of healthy controls. IgGs from IgAN patients recognized HR-GalNAc-BSA but not HR-BSA. These experiments thus confirmed that IgG autoantibodies from IgAN patients recognize terminal GalNAc on IgA1 HR (140, 182).

To better understand at a molecular level the nature of IgG autoantibodies specific for Gd-IgA1, panels of monoclonal IgG autoantibodies were cloned and characterized. EBV-immortalized IgG-secreting lymphocytes derived from peripheral blood of patients with IgAN and healthy controls were generated and, using limiting dilutions, single-cell clones producing IgG specific for Gd-IgA1 were isolated (182). Using single-cell RT-PCR, variable regions of heavy and light chains were amplified, cloned, and sequenced. Selected paired variable regions of heavy and light chains were also cloned and expressed as recombinant IgG, and binding to Gd-IgA1 was assessed. These experiments confirmed and extended previous observations that IgG autoantibodies bound to Gd-IgA1 and that such binding required terminal GalNAc. Moreover, sequence analysis of variable regions of heavy chains of IgG autoantibodies and comparison of the binding of the IgG to Gd-IgA1 pointed out some interesting features. For example, complementarity determining region 3 (CDR3) of variable region of heavy chain tended to be longer in IgG autoantibodies from patients with IgAN compared to that of IgG from healthy controls. Furthermore, a Ser residue was in the third position of CDR3 of autoantibodies in six of the seven studied patients with IgAN. In contrast, IgG from six healthy controls had Ala in that position (182). These observations thus implicated Ser residue in CDR3 in binding of Gd-IgA1. Recombinant IgG from a patient with IgAN was generated by site-directed mutagenesis to change the Ser residue in the third position of CDR3 of the heavy chains to Ala. This mutation reduced binding to Gd-IgA1. Conversely, introducing Ser residue in the third position of CDR3 of the heavy chains of IgG from a healthy control increased the binding of the IgG to Gd-IgA1 (182). Recent study has shown that Ser in CDR3 in the heavy chains of IgG autoantibodies originates from somatic mutations rather than from rare variants of *VH* genes (183).

## Engineered Immune Complexes Consisting of Galactose-Deficient IgA1 and Assessment of Their Biological Activity

It has been observed that levels of IgA1-containing immune complexes in patients with IgAN correlated with clinical and histological activity (27). It was later clarified that such complexes consist of Gd-IgA1 bound by antiglycan antibodies (49, 184, 185). To study biological activities of IgA1-containing immune complexes, a model of cultured primary human mesangial cells has been used (186). With this approach, it was shown that Gd-IgA1-containing immune complexes from patients with IgAN bound to the cells more efficiently than did uncomplexed IgA1 or immune complexes from healthy controls (53, 91). Moreover, large-molecular-mass complexes from sera of patients with IgAN stimulated cellular proliferation and production of cytokines (*e.g*., IL-6 and TGF-β) and components of extracellular matrix (50–52, 61, 91, 95, 187–192). The role of IgA1-containing immune complexes in these activities is confirmed by the fact that IgA1-depleted fractions are devoid of such stimulatory activities (50, 91, 95). Consistent with this finding, when sera of IgAN patients are supplemented with small quantities of polymeric Gd-IgA1, new IgA1-containing immune complexes are formed and, thus, the amount of stimulatory large-molecular-mass immune complexes increases (50, 91). These complexes contain IgG in addition to IgA1, just as do the native complexes (61, 95). In contrast, uncomplexed polymeric Gd-IgA1 or smaller immune complexes do not induce proliferation of cultured primary human mesangial cells.

Supplementation of Gd-IgA1 to serum from IgAN patients formed pathogenic immune complexes (50, 95), indicating an excess of antiglycan antibodies against Gd-IgA1. Following this approach, a new protocol for *in vitro* production of biologically active IgA1-containing immune complexes was developed. Cord blood serum, known to contain IgG but no other immunoglobulins, with high levels of antiglycan IgG was used to bind to Gd-IgA1 myeloma proteins to form immune complexes. Formation of biologically active immune complexes that stimulated cellular proliferation of cultured primary human mesangial cells required Gd-IgA1, antiglycan IgG antibody, and a heat-sensitive serum factor (193).

This model of formation of engineered immune complexes was later enhanced by using recombinant IgG specific for Gd-IgA1 from a patient with IgAN (182) with serum as the source of other factor(s) (193). Notably, these engineered immune complexes stimulate signaling in cultured primary human mesangial cells and increase cellular proliferation in a similar fashion as with native IgA1-containing complexes in sera of patients with IgAN (194, 195).

## Composition of Immune Complexes Consisting of Galactose-Deficient IgA1

In IgAN, complement C3 frequently colocalizes with IgA in mesangial immunodeposits (2, 6, 196) and is also present in IgA1-containing circulating immune complexes of patients with IgAN (28). Moreover, a deletion of *CFHR1* and *CFHR3* genes encoding complement factor H-related factors 1 and 3, the factors involved in the regulation of factor H (197–199), protects against the occurrence of IgAN (8, 150, 179). These observations underscore the contribution of the complement alternative pathway (AP) to pathophysiology of the disease [recently reviewed Ref. (200)]. Using the abovementioned model of engineered immune complexes and targeted proteomic and immunologic analyses, complement C3 products associated with these Gd-IgA1–rIgG complexes were studied (201). Proteomic analysis revealed C3 α and β chain elements in the active large-molecular-mass Gd-IgA1–rIgG immune complexes and only low amounts of β chain in corresponding fractions in a negative control (serum only, not supplemented with Gd-IgA1 or rIgG). Amino-acid sequence by mass spectrometric analysis of specific bands from SDS-PAGE identified iC3b, C3c, and C3dg in the Gd-IgA1–rIgG immune complexes (201). Presence of these C3 fragments was confirmed by immunoblotting. Thus, biologically active Gd-IgA1–rIgG complexes activate complement C3 *in vitro* and associate with C3 degradation fragments. The observed C3 components (iC3b, C3c, and C3d) result from the combined action of factors I and H, suggesting a critical role of regulators in activation of the complement AP in IgAN (200). Thus, (1) addition of serum to Gd-IgA1 bound by anti-Gd-IgA1–IgG autoantibody results in dose-dependent formation of pathogenic immune complexes that activate cultured human mesangial cells and (2) stimulatory immune complexes contain activated C3 products. The relatively small size of these C3 fragments in the nephritogenic immune complexes (molecular mass ~800 kDa) and the association of these C3 fragments with Gd-IgA1–IgG immune complexes suggest direct binding of C3 and activation of the alternative complement pathway in this *in vitro* model of IgAN immune complexes (201).

## Role of Complement in IgA Nephropathy

The role of complement in the pathogenesis of IgAN has been suspected since 1980s, based on the commonly observed mesangial codeposition of C3 with IgA (2, 200, 202).

### Overview of Complement Activation

**Figure 5:**
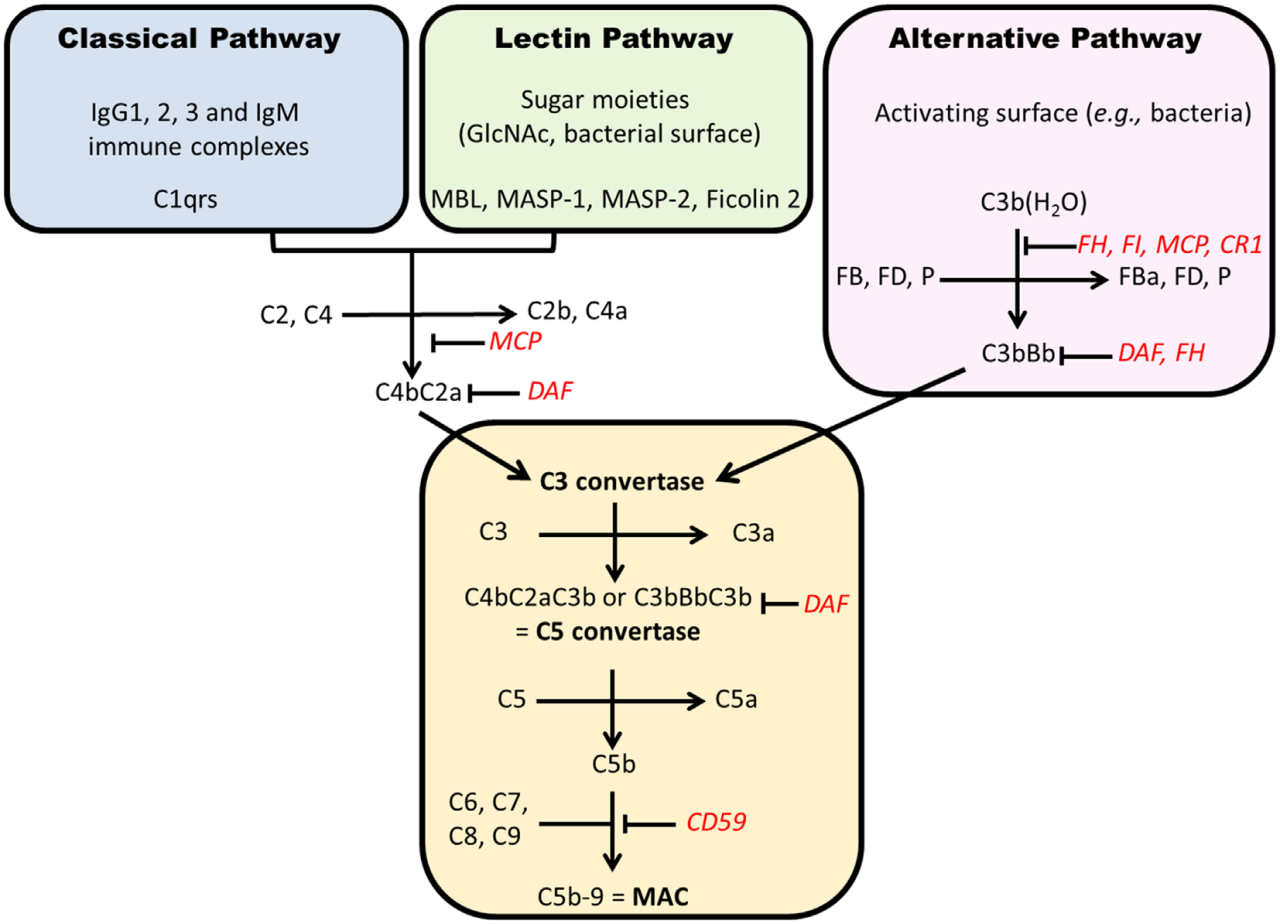
**Complement activation pathways**. Each pathway results in formation of a C3 convertase that, after addition of C3b, becomes a C5 convertase. The generation of C5b starts the formation of membrane attack complex (C5b–9). Regulatory factors are in red. CR1, complement receptor 1; FD, factor D; MAC, membrane attack complex; MCP, membrane cofactor protein; P, properdin; DAF, decay accelerating factor; MBL, mannan-binding lectin; MASP, MBL-associated serine proteases.

**Figure 6:**
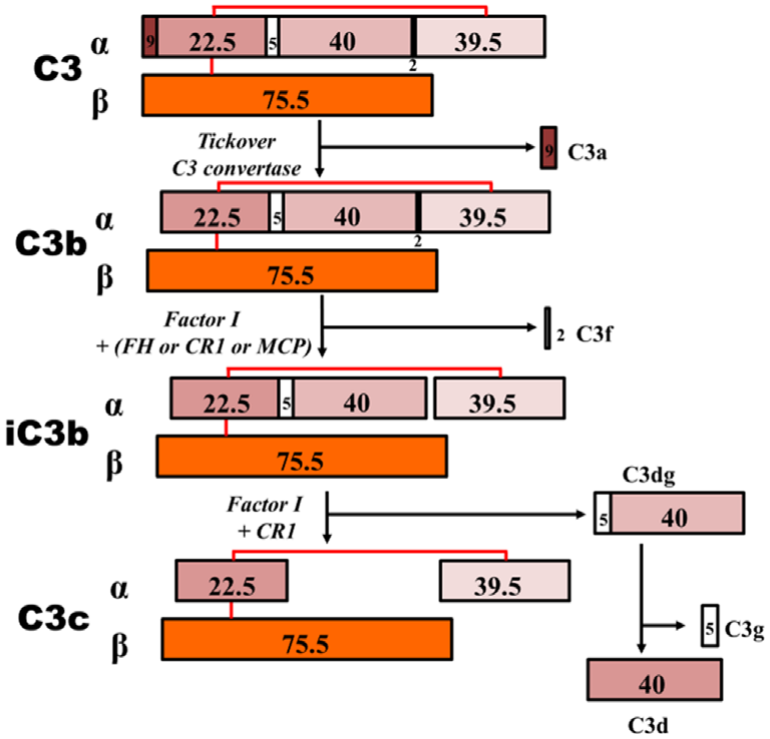
**C3 proteolytic cascade**. The hydrolysis of C3 leads to release of activation products C3a – an anaphylatoxin – and C3b. C3b binds activating surfaces, such as a bacterial cell wall, triggering the alternative pathway cascade. This activation is controlled by regulator molecules, such as FI, FH, and complement receptor 1 (CR1), that degrade C3b into products that cannot contribute to the formation of the C5 convertase (iC3b, C3c, C3dg, and C3d). Detection of these inactive breakdown products is considered evidence of activation of C3. The numbers, in kilodaltons, represent the molecular masses of the corresponding polypeptides. MCP, membrane cofactor protein.

Complement can be activated through three pathways (Figure 5). The classical pathway (CP) is initiated by the recognition of some IgG subclasses (IgG1 and IgG3, and IgG2 to a lesser extent) and IgM by C1q. C1q then binds C1r and C1s and cleaves successively C2 and C4 to form C4b2a complex, a C3 convertase. The AP is activated continuously by spontaneous hydrolysis of C3, exposing an unstable thioester bond and changing C3 conformation to allow its interaction with complement factor B, forming C3(H_2_O)Bb, which cleaves C3 into C3a and C3b (Figure 6) (203). This process is tightly controlled by AP regulatory proteins, such as complement factors I and H, and DAF. Without these regulators, especially on an activating surface (such as a bacterial cell-wall glycan), an amplification loop develops in the presence of factor D and properdin, leading to the accumulation of C3bBb, the AP C3 convertase. The third pathway, the “lectin pathway,” is activated by some sugar moieties, such as mannose or glucosamine on the surface of bacterial cell walls, through interaction with mannan-binding lectin (MBL). The activation process is thereafter similar to the CP to generate C4b2a. Finally, C3 convertase cleaves C3 into C3b that is added to the complex to form C5 convertase. This complex cleaves C5 into C5a and C5b. The latter product binds C6, C7, C8, and C9 (C5b–9) to form the terminal complement complex that can insert into cell-membrane lipid bilayers. This final process can lead to cell lysis or, more commonly in nucleated cells, cellular stress (sublytic complement attack) (204, 205).

### Involvement of Complement Pathways

Alternative pathway is considered an important player in the pathogenesis of IgAN. First, key AP components are codeposited with IgA in the glomerular mesangium. C3 is detected in the immunodeposits in kidney tissue in up to 90% of cases (206–208) as well as properdin (75–100%) and factor H (30–90%) (202, 209, 210). Plasma concentrations of C3 inactivation products (iC3b and C3d) are elevated, reflecting increased production of C3b (211–213). IgA can activate AP *in vitro*, especially while immobilized on a surface in a polymeric form (214, 215).

More recently, GWAS (150, 179) identified a single nucleotide polymorphism (SNP) at position 1q32 in *factor H* gene that was strongly protective against IgAN (odds ratio 0.74 for one allele and 0.55 for two alleles). This SNP was in total linkage disequilibrium with the large deletion of *complement factor H-related genes 1 and 3* (*CFHR1* and *CFHR3*), positioned downstream of *factor H* gene. The copy number association study confirmed the protective impact of this deletion on the risk to develop IgAN. Products of these genes are also AP regulatory proteins that can bind C3 in a similar way as with factor H (216). However, these proteins are less efficient than factor H to regulate AP, such that their absence could lead to a stronger factor H-mediated AP inhibition (198). A recent study has shown that *CFHR1* and *CFHR3* deletion was associated with higher serum levels of factor H and C3, lower serum C3a levels, and less C3 mesangial deposition in Chinese patients with IgAN (199).

The lectin pathway has been examined as a potential mediator for IgAN severity and/or progression of the disease (124, 217, 218). *In vitro* activation of this pathway by polymeric immobilized IgA certainly occurs (219). Several studies have confirmed the negative prognostic impact of the mesangial codeposition of lectin pathway elements, including MBL, MBL-associated serine proteases (MASP-1 and MASP-2), L-ficolin, C4d, and C4-binding protein (220, 221).

The CP is not considered to be a significant player in IgAN, as IgA cannot activate it and actually hinders its activation by IgG (215). C1q is usually missing in IgAN kidney biopsies (<10%, as trace) (207, 222), and the presence of C4 is more representative of lectin pathway activation (220).

The terminal complement complex is commonly codeposited with IgA (210, 223), and its urinary excretion is increased (224). Sublytic C5b–9 can induce mesangial stress, potentially leading to the elevated production of fibronectin, TGF-β, and IL-6 (205, 225). Podocytes can also be severely affected by C5b–9 that can cause cell injury (204, 226).

### Site of Complement Activation

The elevated levels of plasma C3 breakdown products in IgAN patients suggest a soluble-phase activation of the AP. Similarly, a model of mixed IgA–IgG complexes supported this conclusion and indicated that C3 activation required IgG (227). Recently, proteomic analyses of patients’ circulating immune complexes, as well as engineered *in vitro* complexes (formed with polymeric Gd-IgA1, antiglycan IgG, and IgA/IgG-depleted normal serum), revealed cleavage products in high-molecular-mass fractions isolated by size-exclusion chromatography (201). Thus, IgAN immune complexes can act as a surface for AP activation, leading to cleavage of C3 into C3b and thereafter to factor I-dependent inactivated C3 products (iC3b, C3c, C3d, and C3dg).

C3 glomerulonephritis illustrates that AP activation leading to mesangial deposition of C3 products can induce a mesangioproliferative disorder by itself, without significant deposition of immunoglobulins (228). Mesangial cells are potent players in complement-driven glomerular inflammation. They produce factor H and, under inflammatory conditions (IL-1 and TNF-α), express C3 (229). Mesangial cells can express C3 after stimulation by Gd-IgA1-containing immune complexes (230). C3a, an anaphylatoxin produced by the cleavage of C3, can induce cultured human mesangial cells to switch to a secretory phenotype that leads to increased production of mesangial extracellular matrix elements (231).

### Impact on Disease Activity and Progression

Complement consumption and deposition in patients with kidney disease can be assessed with serum, urine, and kidney biopsy specimens. A decreased serum C3 level has been proposed as a disease activity biomarker in several studies from Asia. Serum IgA/C3 ratio has also been associated with IgAN severity (232, 233). A European pediatric study showed a positive correlation of IgA/C3 ratio with clinical- and Oxford classification-based kidney tissue injury (234). Whether plasma factor H level could be a reliable disease activity biomarker remains uncertain, as findings of other studies were inconsistent (235, 236).

Urinary excretion of complement elements has also been evaluated, mostly in Asia. Two studies showed greater excretion of factor H (237) and C5b–9 (224) compared to healthy controls, but without a disease-control group with proteinuria.

The deposition of complement elements in glomeruli could also be a valuable tool to predict IgAN progression. The intensity of mesangial C3 deposition was associated with worse clinical outcome (238, 239). Finally, activation of lectin pathway leading to C4d deposition in IgAN predicted worse outcomes in three retrospective studies (221, 240, 241).

## Activities and Catabolism of IgA1-Containing Circulating Immune Complexes

Size and composition of immune complexes determine biological activities (50, 71, 95, 193). Based on the size, circulating IgA1-containing immune complexes in IgAN patients can be divided into two groups: immune complexes with high molecular mass (>800 kDa) and immune complexes with low molecular mass (≤800 kDa). Notably, the high-molecular-mass complexes activate cultured human mesangial cells, as indicated by cellular proliferation and overproduction of cytokines and components of extracellular matrix (50, 95). In contrast, the low-molecular-mass complexes exhibit an inhibitory effect (95). Circulating immune complexes with higher content of Gd-IgA1 have enhanced capacity to induce proliferation of mesangial cells, whereas complexes without Gd-IgA1 or Gd-IgA1 alone do not have proliferative effects (50). Stimulation of proliferation of mesangial cells by immune complexes containing Gd-IgA1 was confirmed by experiments with *in vitro*-formed immune complexes (193).

Stimulatory Gd-IgA1-containing complexes induce not only cellular proliferation but also production of laminin, a protein component of extracellular matrix (95). Similarly, production of laminin was increased by stimulation with TGF-β in a murine mesangial-cell model (242). Large-molecular-mass complexes bind to CD71 and activate mitogen-activated protein kinase/extracellular-signal-regulated kinase (MAPK/ERK) pathway (243). This cellular activation alters crosstalk between mesangial cells and podocytes through TNF-α and TGF-β. These cytokines are released from mesangial cells in elevated amounts and induce expression of nephrin, erzin, and podocin in podocytes (191, 192). Furthermore, elevated production of TGF-β could contribute to glomerular fibrosis by enhancing expression of profibrotic genes driving accumulation of extracellular matrix. TGF-β increases expression of profibrotic connective tissue growth factor (CTGF) *via* sphingosine 1-phosphate receptor 5 (S1P_5_) on cultured human mesangial cells (244, 245).

### Mesangial Receptors for IgA1-Containing Immune Complexes

It is not known which receptor(s) on mesangial cells plays a key role in binding to Gd-IgA1-containing immune complexes and activation of human mesangial cells. Myeloid IgA Fc receptor (CD89) and ASGP-R are not expressed on human mesangial cells (53, 246–249). Additional details on Fc receptors, including those on mesangial cells, can be found in a recent review with an extensive list of references (250). Currently, it is thought that the main receptor is CD71, known as transferrin receptor. CD71 is highly expressed in glomeruli of IgAN patients, and its localization correlates to deposits of IgA (251, 252). Moreover, studies using mice expressing human IgA1 heavy chain and human CD89 indicated that complexes of IgA1–sCD89 could initiate an autoamplification process involving overexpression of transferrin receptor 1 (TFR1) and transglutaminase 2 (TGase2). Involvement of sCD89–IgA1 complexes and participation of TFR and TGase2 explain an alternative mechanism of mesangial-cell activation (253). Adding to the complexity, other receptor candidates from a family of integrins (integrin α1/β1 and integrin α2/β1) also bind IgA1 on mesangial cells (254).

Taken together, local inflammation, cellular proliferation, and increased production of extracellular matrix components by mesangial cells activated by IgA1-containing complexes considerably impact glomerular function, leading to hematuria and proteinuria. Without disease-specific therapy, many patients progress to end-stage renal disease and require renal replacement therapy.

## Animal Models

Small-animal models of IgAN can be very helpful in studies of various aspects of disease pathogenesis or testing efficacy of new therapeutic approaches. However, development of such models for IgAN has been hindered because only humans and hominoid primates have IgA1 with its *O*-glycans, a pivotal component in the pathogenesis of human disease. For example, mice have only one subclass of IgA and it resembles human IgA2 (255). However, several different models have been developed that may elucidate various specific aspects of IgAN (256) (Table 1).

**Table 1:** **Selected animal models of IgAN**.

Model	Key features and comparisons with human IgAN
Spontaneous ddY mouse	A spontaneous model of IgAN with mesangial deposits of murine IgA associated with glomerular injury. This model has a high degree of variability in the age of onset and severity of the disease, due to the heterogeneous genetic background (257)
High-IgA strain of ddY mouse (HIGA)	Established by interbreeding of ddY strains with high serum levels of murine IgA. HIGA mice have high serum IgA levels; however, serum IgA levels are not associated with the severity of glomerular injury and disease incidence (258, 259)
Grouped-ddY mouse	Includes the early-onset group of ddY mice intercrossed over 20 generations, in which the development of IgAN includes mesangial deposits of murine IgA. Glomerular injury and proteinuria develop within 8 weeks of age. This model allows genetic analysis and studies of the pathogenesis involving IgA–IgG immune complex formation (260)
Spontaneous IgAN in marmosets	Marmosets in captivity are highly susceptible to a wasting syndrome that is apparently associated with IgA antigliadin antibodies and IgA-containing circulating immune complexes that deposit in the glomerular mesangium. Notably, this syndrome disappears after gluten is withdrawn from the diet. It is yet to be determined whether this syndrome may present a suitable animal model for human celiac disease and/or IgAN (261)
β1, 4-galactosyltransferase-I-deficient mouse	These mice have a gene for a galactosyltransferase knocked-out and exhibit high serum levels of IgA with elevated portions of polymeric IgA. These mice have mesangial deposits of murine IgA and the *N*-glycans are deficient in galactose (262)
Human BAFF-transgenic mouse	Overexpression of human BAFF in mice results in elevated serum levels of murine IgA. Fatal glomerulonephritis is associated with mesangial deposits of IgA (135)
IgA1-CD89-transgenic mouse	Complexes of transgenic human IgA1 heavy chain-containing IgA with transgenic human-soluble CD89 deposit in the mesangium and induce hematuria and proteinuria. These mice develop mesangial IgA deposits, glomerular and interstitial macrophage infiltration, mesangial matrix expansion, hematuria, and mild proteinuria. Some studies question whether transgenic CD89 in this mouse model is involved in a similar manner as in humans (253, 263)
Passive mouse model of IgAN	Immunodeficient mice (*e.g*., SCID mice) are injected with preformed complexes of human Gd-IgA1 bound by antiglycan human IgG. The complexes of human immunoglobulins deposit in the glomerular mesangium with murine C3 co-deposits and induce mesangial proliferation, hematuria, and proteinuria. Human IgA1 autoantigen (Gd-IgA1) and IgG autoantibodies are used, but the model requires several injections of preformed complexes (264, 265)

### Spontaneous Models

Spontaneous IgAN models include ddY mice, high-IgA (HIGA) mice, early-onset-grouped ddY mice (257–260), and marmosets’ wasting syndrome. The last model is associated with IgA antigliadin antibodies and IgA-containing circulating immune complexes that deposit in the mesangium (261). The ddY mouse is a model of spontaneous murine IgAN based on development of glomerulonephritis associated with mesangial deposition of IgA with co-deposits of IgG, IgM, and C3 (257). Based on the age of disease onset, ddY mice are categorized as early-onset, late-onset, and quiescent (*i.e*., no glomerulonephritis) phenotypes and are amenable to genomic analyses (259). GWAS identified four genetic susceptibility loci (*D1Mit216*, *D1Mit16*, *D9Mit252*, and *D10Mit86*) linked with the early-onset phenotype (259). The HIGA mouse strain was generated by interbreeding ddY mice with high serum IgA levels (258). However, serum IgA levels in HIGA mice were not associated with severity or incidence of disease (259). A more informative mouse model was developed by intercrossing early-onset ddY mice (260). These early-onset-grouped ddY mice develop proteinuria by 8 weeks of age and renal failure at 24 weeks of age. The grouped early-onset ddY mice show severe glomerular and tubulointerstitial lesions, characterized by mesangial proliferation, mesangial matrix expansion, and tubulointerstitial cellular infiltration. This model may provide useful insights into the pathogenesis of disease, to include identifying susceptibility genes, defining the role of IgA polymorphisms and IgA-containing immune complexes, and assessing the gender difference in progression of disease.

### Models with Altered Genes

Knock-out and transgenic animal models include β1,4-galactosyltransferase-I-deficient mice, human BAFF-transgenic mice, and IgA1-CD89-transgenic mice (135, 253, 262, 263). The β1,4-galactosyltransferase-I-deficient mice show semi-lethality before weaning due to growth retardation and reduced inflammatory responses. The surviving β1,4-galactosyltransferase-I-deficient mice developed similarly as did control mice. However, starting from 10 weeks of age, the β1,4-galactosyltransferase-I-deficient mice developed an IgAN-like disease associated with high serum IgA levels with greater portions of polymeric IgA. Histological examination of kidneys showed IgA deposition, expanded mesangial matrix, and electron-dense deposits in the paramesangial regions.

The model of BAFF-transgenic mice showed high serum IgA levels with increased portions of polymeric IgA and IgA deposition in the glomeruli but only in mice with microbiota (not in mice without microbiota) (135). This finding emphasized the role of microbiota in driving IgA responses in species/individuals with a specific genetic background. Another transgenic model includes mice producing IgA consisting of heavy chains of human IgA1 with murine light chains. In mice with a transgene to produce the soluble fragment of human CD89, circulating IgA–CD89 complexes form (253, 263). These transgenic mice develop mesangial IgA deposits, glomerular and interstitial macrophage infiltration, mesangial matrix expansion, hematuria, and mild proteinuria. However, follow-up studies have raised questions whether CD89 is involved in a similar manner in human IgAN, as mice do not have a homolog of human CD89 (266, 267).

### Passive Model

We have recently developed a passive mouse model of IgAN based on injection of SCID mice with preformed immune complexes consisting of human Gd-IgA1 bound by antiglycan antibodies (264, 265). These Gd-IgA1–IgG complexes deposit in the glomerular mesangium with murine C3 and induce mesangial proliferation, hematuria, and proteinuria. This model further supports the key roles of aberrant *O*-glycosylation of IgA1 and the corresponding autoantibodies specific for these IgA1 glycoforms in formation of glomerular immunodeposits in IgAN.

## Four-Hit Model of IgA Nephropathy Pathogenesis

**Figure 7:**
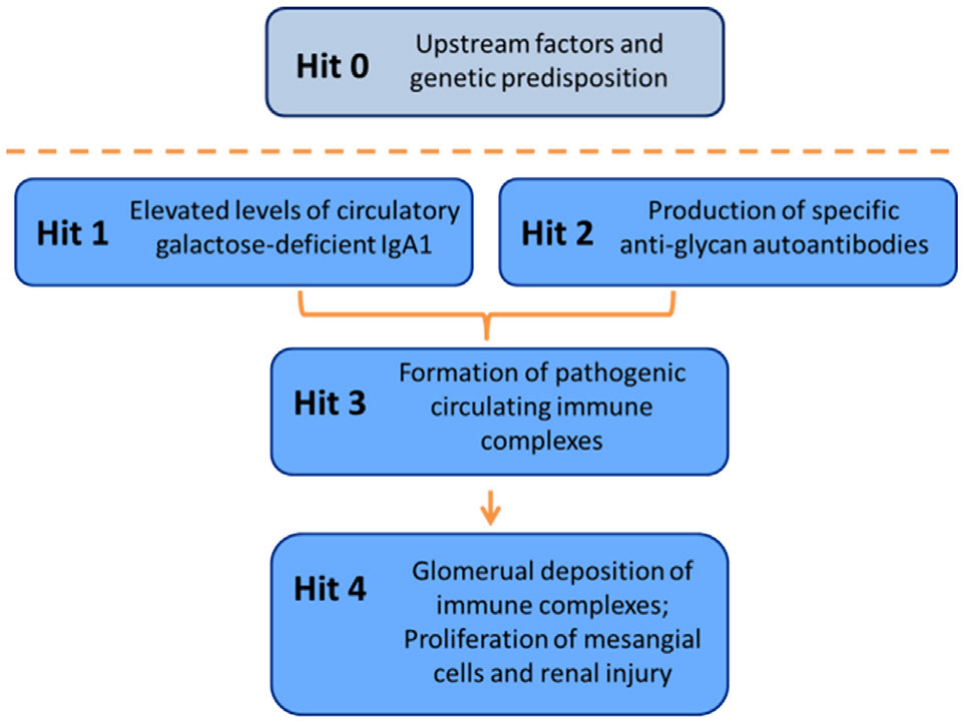
**Multi-hit hypothesis for pathogenesis of IgAN**. Several processes are involved in development of IgAN. Circulatory Gd-IgA1 (Hit 1) is recognized by Gd-IgA1-specific autoantibodies (Hit 2) that leads to formation of pathogenic Gd-IgA1-containing circulating immune complexes (Hit 3). Some of these complexes reach the renal glomeruli to bind to mesangial cells and activate them, thereby inducing renal injury (Hit 4).

Clinical and laboratory research during recent years has led to a widely accepted definition of IgAN as an autoimmune disease with a complex multistep, also called multi-hit, pathogenetic process (Figure 7) (173). Specifically, circulatory Gd-IgA1 in patients with IgAN (Hit 1) is recognized by autoantibodies of IgG and/or IgA isotype (Hit 2). Subsequently, IgA1–IgG and IgA1–IgA1 immune complexes are formed (Hit 3) that contain additional proteins, including components of complement system (200, 201). Some of these immune complexes ultimately deposit in the glomerular mesangium to activate mesangial cells and induce renal injury (Hit 4) (38, 61, 173). An alternative hypothesis has been proposed to suggest that aberrantly glycosylated IgA1 accumulates in the mesangium as lanthanic deposits that are later bound by newly appearing autoantibodies, resulting in the *in situ* formation of immune complexes (268). The immune deposits stimulate the mesangial cells to proliferate and overproduce components of extracellular matrix, cytokines, and chemokines. Some of these cytokines may cause podocyte injury to induce proteinuria (191, 269). Complement likely plays a role in the formation and activities of these complexes in the circulation as well as in those that may be formed *in situ* [for review, see Role of Complement in IgA Nephropathy above, and Refs (230) and (200)]. Moreover, some of the hits in the pathogenesis may be modulated or controlled by various environmental and genetic factors [for review, see Ref. (270)].

Multiple other publications and findings lend credence to the multi-hit hypothesis on the pathogenesis of IgAN. For example, serum levels of Gd-IgA1 may predict disease progression (32), and serum levels of IgG and/or IgA autoantibodies specific for Gd-IgA1 correlate to disease severity and may also predict disease progression (182, 271). Moreover, serum levels of Gd-IgA1, IgG autoantibodies, and IgA1–IgG immune complexes predict disease recurrence in renal allografts (272).

Progress in the clinical and laboratory studies of IgAN has fueled a paradigm-shifting hypothesis on the autoimmune nature of the disease and identified some of the associated genetic factors (270). The multi-hit hypothesis not only describes the pathogenetic steps of IgAN but also serves as a “blueprint” for identifying targets of future disease-specific therapy and developing key biomarkers of the disease.

## Biomarkers of IgA Nephropathy

Clinical and laboratory studies in the last several years have identified several potential biomarkers for IgAN. It is hoped that some of these candidate markers can be developed into clinical assays to aid in the diagnosis, prognosis, patient stratification, monitoring of disease progression, and assessment of responses to treatment. Below, we briefly outline some of the candidate markers and also mention prospects for the development of disease-specific therapy.

### Genetic/Genomic Biomarkers

Involvement of genetic factors in IgAN was first recognized through the discovery of familial forms of the disease (273). Specific loci and genes were later identified through linkage studies and GWAS [for review, see Ref. (274)]. Multiple susceptibility alleles have been identified by GWAS in cohorts from Europe, North America, and East Asia (179, 275–277). Disease susceptibility is affected by common variations in genes involved in antigen processing and presentation as well as in the mucosal defense system and alternative complement pathway. These findings further support an autoimmune nature of IgAN. GWAS data revealed that common genetic variants influence the risk of IgAN and suggest a multilocus adaptation process, possibly related to the variation in local pathogens across world populations (179). Moreover, serum levels of Gd-IgA1, the key autoantigen in IgAN, are genetically codetermined (152). Multiple risk and protective alleles among these disease-associated genes have been uncovered, and the cumulative number of risk alleles has been linked to the age of disease onset (179). However, additional genomic studies are needed to better define major genetic factors and their variants and to enable development of future individualized genetic/genomic approaches.

### Serum Biomarkers

A better understanding of the causes of IgAN through combined clinical, biochemical, and molecular studies will identify candidates for developing disease-specific biochemical biomarkers. Candidate biomarkers include serum levels and/or specific characteristics of the autoantigen (Gd-IgA1), levels of autoantibodies specific for Gd-IgA1, and levels and/or specific characteristics of immune complexes consisting of IgG autoantibody bound to Gd-IgA1 (181, 272, 278) (Table 2). These biomarkers, whether used individually or in combination as panels, may have diagnostic and/or prognostic significance and would support future testing of disease-specific therapeutic approaches.

**Table 2:** **Candidate biomarkers and disease-specific approaches for treatment of IgAN**.

Pathogenic step	Candidate biomarkers	Potential approaches and targets of disease-specific therapy
Elevated production of Gd-IgA1	Serum level of Gd-IgA1 (lectin or antibody ELISA) IgA1 hinge-region *O*-glycopeptide profiles (mass spectrometric analysis)	Reduce production of Gd-IgA1 Manipulate enzyme expression in IgA1-producing cells Reduce number of cells secreting Gd-IgA1
Production of autoantibodies specific for Gd-IgA1	Serum levels of autoantibodies (IgG and IgA) specific for Gd-IgA1	Reduce production of autoantibodies specific for Gd-IgA1 Deplete cells producing the autoantibody Manipulate affinity maturation of autoantibodies to reduce affinity for the autoantigen Remove the autoantibodies from circulation
Formation of pathogenic IgA1-containing immune complexes	Circulating IgA-containing immune complexes Specific components of circulating immune complexes	Block immune complex formation and enhance their removal from circulation and catabolism Block epitopes of autoantigen (Gd-IgA1) by non-crosslinking antibodies Block autoantibodies by an epitope-containing glycopeptide or glycomimetic Block activation of complement
Glomerular deposition and injury	Complement components and their degradation products Novel markers of glomerular injury Urinary immune complexes Urinary peptidomic profiles	Block activation of mesangial cells Reduce complement activation *in situ* Block binding of IgA1-containing immune complexes to mesangial cells Block mesangial-cell signaling induced by IgA1-containing immune complexes

### Urinary Biomarkers

IgAN is diagnosed based on evaluation of a renal biopsy specimen. Laboratory screening for the possible presence of the disease include assessment of proteinuria and hematuria. These measurements are not disease-specific and, thus, there have been numerous efforts to identify urinary markers specific for IgAN (279–281). For example, urinary concentrations of several cytokines related to cellular proliferation were evaluated as potential markers of histopathologic glomerular and tubulointerstitial changes. Urinary IL-6 levels were elevated in patients with glomerulonephritis; however, the results did not define the type of primary glomerulonephritis (282). Nonetheless, urinary excretion of IL-6 predicted long-term renal outcome in patients with IgAN (283), and excretion of IL-6 and epidermal growth factor (EGF) has been correlated with degree of tubulointerstitial damage that itself predicts a poor long-term outcome (3, 4). Based on these results, the ratio of urinary IL-6/EGF was proposed as a prognostic marker for the progression of renal damage (284). In addition, urinary levels of monocyte chemotactic peptide-1 (MCP-1) and IL-8 correlated to tubulointerstitial damage (285, 286). However, cytokine/chemokine excretion again did not distinguish the specific types of glomerulonephritis. Other potential markers that have been evaluated include urinary α-1 antitrypsin in the α-1-globulin fraction (287) and urinary heparan sulfate (288), both of which were significantly higher in patients with IgAN. Urinary IgA concentrations are higher in patients with IgAN than in healthy individuals or in patients with other renal diseases and correlate with proteinuria (289). Immune complexes consisting of Gd-IgA1 and IgG were detected in the urine of patients with IgAN (290), but the prognostic value has not been defined. In contrast, excretion of the membrane attack complex was elevated in patients with membranous nephropathy (291) but not in patients with IgAN (292). Another study showed a correlation between glomerular filtration rate, urinary immunoglobulin excretion, and pathological grading of renal biopsies in patients with HSP with nephritis (293).

In addition to intact proteins, urine contains naturally occurring fragments (peptides) derived from serum and renal tubular or glomerular proteins (280, 294–301). Analysis of urinary peptides may offer an opportunity to develop a non-invasive and unbiased diagnostic tool without *a priori* assumptions as to the pathogenesis of disease (281, 300–305). Initial studies using urinary peptidomic techniques indicated the potential to differentiate patients with IgAN from patients with other glomerular diseases (302, 306).

Although many reports on urinary proteins and peptides demonstrated differential amounts of some proteins and protein complexes as well as peptides in the urine of patients with IgAN, none of the tests has been used in a routine clinical laboratory. It is hoped, however, that future studies will provide markers useful both for diagnosis and therapeutic monitoring of this disease (281, 296, 300, 301, 307).

## Treatment

As outlined in the previous sections, Gd-IgA1-containing immune complexes are considered to be a critical factor in the pathogenesis of IgAN. Theoretically, any intervention that would reduce production of Gd-IgA1 or autoantibodies specific for Gd-IgA1, block formation of the IgA1–IgG complexes, or otherwise reduce levels of pathogenic immune complexes would constitute effective disease-specific treatment. Similarly, any approach that would block activation of mesangial cells by the pathogenic IgA1–IgG complexes would be desirable. Examples of such approaches are listed in Table 2, and more details can be found in recent reviews (6, 180, 308). It is hoped that studies that discover the molecular defects of IgA1, the mechanisms of induction of the autoantibodies specific for Gd-IgA1, the composition and biological activities of the immune complexes, and the signaling pathways for activation of mesangial cells and glomerular injury will lead to disease-specific therapy.

## Conclusion

Accumulated knowledge indicates that IgAN, the most common primary glomerulonephritis in the world, is an autoimmune disease driven by formation and glomerular deposition of IgA1-containing immune complexes. Currently, there is no disease-specific therapy, and many patients with IgAN progress to end-stage renal disease. The diagnosis of IgAN is established by determination of IgA as the dominant or codominant immunoglobulin in glomeruli. The IgA in glomerular deposits is exclusively of the IgA1 subclass and is enriched for glycoforms deficient in galactose on the hinge-region *O*-glycans. Multiple studies led to a hypothesis for a multi-hit pathogenetic process with contributing genetic and environmental components. In this process, circulatory Gd-IgA1 is recognized as an autoantigen by IgG or IgA autoantibodies, resulting in the formation of immune complexes. Some of these circulating complexes deposit in glomeruli, activate mesangial cells, and induce glomerular injury through cellular proliferation and overproduction of components of extracellular matrix and cytokines/chemokines. Glycosylation pathways associated with production of the autoantigen and the unique characteristics of the corresponding autoantibodies in patients with IgAN leading to the formation of pathogenic immune complexes have been uncovered, and genetic factors associated with IgAN have been identified. Complement plays a significant role in the formation and nephritogenic activities of these complexes; complement activation likely occurs systemically on IgA1-containing circulating immune complexes as well as locally in glomeruli. Multiple new models and approaches have been developed that will lead to a better understanding of the molecular mechanisms and factors involved in formation and activities of pathogenic IgA1-containing immune complexes. It is hoped that the ongoing and future studies will enable development of much needed disease-specific therapy (308).

## Author Contributions

JN and BAJ conceived the general outline, BK, CR, and NM further developed the outline and content. Each author contributed intellectually by writing assigned sections, editing and revising the drafts and proofreading.

## Conflict of Interest Statement

The authors declare that the review was written in the absence of any commercial or financial relationships that could be construed as a potential conflict of interest.
